# Meta‐Analysis Comparing Oral Anticoagulant Monotherapy Versus Dual Antithrombotic Therapy in Patients With Atrial Fibrillation and Stable Coronary Artery Disease

**DOI:** 10.1002/clc.70026

**Published:** 2024-10-07

**Authors:** Mushood Ahmed, Areeba Ahsan, Aimen Shafiq, Raheel Ahmed, Mahboob Alam, Pierre Sabouret, Jamal S. Rana, Gregg C. Fonarow

**Affiliations:** ^1^ Department of Medicine Rawalpindi Medical University Rawalpindi Pakistan; ^2^ Department of Medicine Foundation University Medical College Islamabad Pakistan; ^3^ Department of Medicine Dow University of Health Sciences Karachi Pakistan; ^4^ Department of Cardiology Royal Brompton Hospital London UK; ^5^ Department of Cardiology, National Heart and Lung Institute Imperial College London UK; ^6^ Department of Cardiology Baylor College of Medicine Houston Texas USA; ^7^ Heart Institute and Action Group, Pitié‐Salpétrière Sorbonne University Paris France; ^8^ National College of French Cardiologists Paris France; ^9^ Division of Cardiology Kaiser Permanente Northern California Oakland California USA; ^10^ Division of Research Kaiser Permanente Northern California Oakland California USA; ^11^ Ahmanson‐UCLA Cardiomyopathy Center, Division of Cardiology University of California Los Angeles Los Angeles California USA

**Keywords:** anticoagulant, antiplatelet, atrial fibrillation, coronary artery disease

## Abstract

**Background:**

Oral anticoagulants (OACs) are routinely used for the management of atrial fibrillation (AF) while antiplatelet agents are used in coronary artery disease (CAD). However, data regarding the comparative clinical outcomes of OAC monotherapy versus dual antithrombotic therapy (anticoagulant plus antiplatelet agent) in patients with AF and stable CAD are limited.

**Methods:**

A comprehensive search of major databases including PubMed/MEDLINE, Cochrane Library, and Embase was performed from inception to September 1, 2024 to identify randomized control trials (RCTs) that compared OAC monotherapy with dual antithrombotic therapy in patients with AF and stable CAD. The risk ratios (RRs) were estimated with corresponding 95% confidence intervals (CIs) for all outcomes.

**Results:**

A total of three RCTs reported data for 3945 patients with AF and stable CAD. The mean age of patients was 73.8 (±11.85) years and the mean follow‐up was 22 months. OAC monotherapy was associated with a significantly reduced relative risk of major bleeding (RR: 0.55, 95% CI: 0.32–0.95) compared to dual therapy. The risk of all‐cause death (RR: 0.85, 95% CI: 0.49–1.48), cardiovascular death (RR: 0.84, 95% CI: 0.50–1.41), any stroke event (RR: 0.74, 95% CI: 0.46–1.18), and myocardial infarction (RR: 1.57, 95% CI: 0.79–3.12) remained comparable across the two groups.

**Conclusion:**

OAC monotherapy led to a significant relative risk reduction for major bleeding with similar rates of ischemic events and mortality compared to dual antithrombotic therapy in patients with AF and stable CAD.

## 1 Introduction

Atrial fibrillation (AF) is the most prevalent cardiac arrhythmia which significantly increases the risk of stroke and systemic embolism [1]. The management of AF in patients with stable coronary artery disease (CAD) presents a complex therapeutic challenge [2]. Anticoagulation is essential for preventing stroke in AF [3, 4], while antiplatelet therapy plays a key role in the prevention of recurrent ischemic events in CAD [5]. Consequently, many patients with AF and stable CAD are medically managed with a combination of anticoagulants and antiplatelets, leading to dual antithrombotic therapy (DAT). However, the combination of anticoagulation and antiplatelet therapy increases the risk of bleeding complications [6], raising concerns about the optimal treatment strategy for this patient population. Recent studies have increasingly considered oral anticoagulant monotherapy (OAC) as a viable alternative to DAT in patients with stable CAD beyond 1 year after coronary events or interventions. This approach aims to reduce bleeding risks while maintaining adequate thromboembolic protection.

Some studies have compared the safety and efficacy of OAC monotherapy against DAT in patients with AF and stable CAD [7, 8, 9]. However, these studies have reported varying results, with some favoring monotherapy and others advocating for continued dual therapy. Although these studies have contributed valuable insights, the evidence from randomized controlled trials (RCTs) remains limited and often underpowered. Recently, a large‐scale, multicenter clinical trial has emerged [10], offering robust data that could significantly influence the current understanding of optimal antithrombotic therapy in this subset of patients. This new evidence underscores the necessity of revisiting and refining existing management strategies through a comprehensive meta‐analysis that integrates these latest findings with previous research.

Therefore, this meta‐analysis aims to pool the results of this new trial with existing data to provide a more comprehensive evaluation of OAC monotherapy versus DAT in patients with AF and stable CAD.

## 2 Methods

This systematic review and meta‐analysis has been conducted following the Preferred Reporting Items for Systematic Review and Meta‐Analysis (PRISMA) guidelines [11].

### 2.1 Data Sources and Search Strategy

A search of major databases including PubMed/MEDLINE, Cochrane Library, and Embase was conducted from inception to September 1, 2024 to identify randomized control trials (RCTs) that compared OAC monotherapy with DAT in patients with AF and CAD. The websites of major cardiology journals were also searched to identify relevant articles. The search strategy used was based on the following entry terms: “oral anticoagulant,” “OAC,” “warfarin,” “dabigatran,” “rivaroxaban,” “apixaban,” “edoxaban” AND “dual antithrombotic therapy,” “DAT,” “antiplatelet therapy,” “aspirin,” “clopidogrel,” “ticagrelor,” AND “atrial fibrillation,” “AF” AND “coronary artery disease,” “CAD,” “ischemic heart disease,” “myocardial ischemia.” The detailed search strings are provided in Supporting Information S1: Table S1.

### 2.2 Eligibility Criteria and Outcomes

Studies were considered eligible for inclusion in our systematic review and meta‐analysis if they: (i) were published RCTs comparing OAC monotherapy versus DAT; (ii) included patients with AF (paroxysmal, persistent, or permanent AF) and stable CAD; (iii) evaluated at least one of the efficacy or safety outcome. The primary outcomes were all‐cause death, cardiovascular death, and major bleeding events. The secondary outcomes included stroke (ischemic or hemorrhagic), and myocardial infarction.

### 2.3 Study Selection, Data Extraction, and Bias Assessment

The duplicate records were excluded from the studies identified in the literature search. Two investigators (AA and AS) reviewed the titles and abstracts of the studies. Then a review of full‐texts was performed. A third author (MA) was consulted in the event of any disagreements.

The data extracted from eligible trials included: trial name, publication year, country, sample size, type and dose of monotherapy in the treatment arm, dual therapy in the comparison arm, mean CHA2 DS2‐VASc score, CHADS2 score, HAS‐BLED score, duration of follow‐up, age of patients, males, various risk factors/comorbidities such as diabetes, smoking status, history of MI/ischemia, any history of previous stroke or cerebrovascular disease, the type of AF, and clinical outcomes. We used a pre‐piloted Excel sheet for data extraction.

The risk of bias was assessed in the included RCTs using the Cochrane Risk of Bias (RoB 2.0) tool [12]. The risk was assessed across five domains and the trials were scored as high, with some concerns, or low risk of bias in each domain.

### 2.4 Statistical Analysis

The statistical analysis was performed using R version 4.4.1. The risk ratios (RRs) with 95% confidence intervals (CIs) were used as summary estimates and estimated using the random effects model, which were visually presented in forest plots [13]. The Paule‐Mandel estimator was used for tau^2^ [14]. Heterogeneity was assessed using the Cochrane Handbook of Systematic Reviews of Interventions arbitrary cutoff values for the Higgins *I* ^2^ statistic, also considering the results of the *χ*² test: 0%–40%, low heterogeneity; 30%–60%, moderate heterogeneity; 50%–90%: substantial heterogeneity; 75%–100%, considerable heterogeneity [15]. A secondary analysis was conducted by pooling hazard ratios (HRs) reported by trials. The RRs were calculated for the primary analysis due to inconsistent reporting of HRs by the trials for some analyzed outcomes. Moreover, a leave‐one‐out sensitivity analysis was performed in which each trial was sequentially removed from the calculated summary effect sizes to assess its impact on between‐study heterogeneity and whether any particular trial had a high influence on the summary estimates. A *p*‐value of < 0.05 was considered significant for assessed outcomes.

## 3 Results

The literature review yielded 1016 records. The duplicate studies were removed and two investigators independently screened articles using their titles and abstracts. 509 irrelevant studies were removed and full‐texts of 20 studies were retrieved. After the review of full texts, three RCTs meeting the inclusion criteria were included in the meta‐analysis (Supporting Information S1: Figure S1).

**Figure 1:**
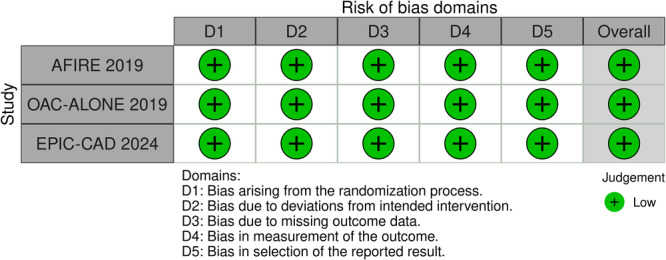
Risk of bias summary for the included RCTs.

The included studies [10, 16, 17] reported data for 3945 patients with AF and stable CAD. 1975 patients received OAC monotherapy and 1970 patients received DAT. The mean age of patients was 73.8 (±11.8) years. Male patients constituted 79.6% of the study sample. The mean follow‐up duration was 22 months. Two studies were conducted in Japan and one in South Korea. The AF and Ischemic Events With Rivaroxaban in Patients With Stable CAD (AFIRE) study [16] compared rivaroxaban monotherapy with rivaroxaban plus a single antiplatelet drug. The Optimizing Antithrombotic Care in Patients With AF and Coronary Stent (OAC‐ALONE) trial [17] compared OAC monotherapy with an oral anticoagulant plus an antiplatelet agent in patients with AF and CAD more than 1 year after stenting. The EPIC‐CAD (Edoxaban vs. Edoxaban with Antiplatelet Agent in Patients with AF and Chronic Stable CAD) trial [10] compared edoxaban monotherapy with endoxaban plus an antiplatelet agent in patients with AF and CAD who had undergone stent implantation or managed medically previously. The selection of the antiplatelet agent was consistent across the trials, with most patients receiving aspirin (85.9% in the OAC‐ALONE trial, 70.2% in the AFIRE study, and 61.8% in the EPIC‐CAD trial). All patients had undergone coronary stent implantation in the OAC‐ALONE trial, in the AFIRE trial 70.6% patients had undergone PCI and 11.3% patients had undergone coronary artery bypass grafting, while in the EPIC‐CAD trial, 65.7% patients had undergone revascularization procedures (PCI or coronary artery bypass grafting) and 34.3% received medical management only. The details are provided in Table 1 and Supporting Information S1: Table S2. A low risk of bias was observed in all included trials (Figure 1).

**Table 1:** Baseline characteristics of included studies and patients.

Trial	Year	Country	Sample size		Type and dose of monotherapy	Dual therapy group	Follow up‐ months	CHA2 DS2‐VASc score^a^	CHADS2 score	HAS‐BLED score	Age‐ mean ± SD		Males‐ n (%)		Paroxysmal AF‐ n (%)		Persistent AF‐ n (%)		Permanent AF‐ n (%)	
			OAC monotherapy	Dual therapy							OAC Monotherapy	Dual therapy	OAC Monotherapy	Dual therapy	OAC Monotherapy	Dual therapy	OAC Monotherapy	Dual therapy	OAC Monotherapy	Dual therapy
AFIRE	2019	Japan	1107	1108	Rivaroxaban 15 mg	Rivaroxaban plus Aspirin or P2Y12 inhibitor	24.1	4	2	2	74.3 ± 8.3	74.4 ± 8.2	875 (79.0)	876 (79.1)	596 (53.8)	580 (52.3)	164 (14.8)	175 (15.8)	347 (31.3)	353 (31.9)
OAC‐ALONE	2019	Japan	344	346	Warfarin or DOAC	Warfarin or DOAC plus Aspirin or clopidogrel	30	4.6	2.5	2	74.9 ± 0.4	75.2 ± 0.4	294 (85.5)	294 (85.0)	158 (45.9)	143 (41.3)	27 (7.9)	23 (6.7)	159 (46.2)	180 (52.0)
EPIC‐CAD	2024	South Korea	524	516	Edoxaban 60 mg once daily	Edoxaban plus Aspirin or a P2Y12 inhibitor	12	4	2	2	71.7 ± 8.0	72.5 ± 8.4	396 (75.6)	406 (78.7)	292 (55.7)	283 (54.8)	232 (44.3)	233 (45.2)	232 (44.3)	233 (45.2)

*Note:* AFIRE, atrial fibrillation and ischemic events with rivaroxaban in patients with stable coronary artery disease; OAC‐ALONE, the optimizing antithrombotic care in patient with atrial fibrillation and coronary stent; EPIC‐CAD, edoxaban versus edoxaban with antiplatelet agent in patients with atrial fibrillation and chronic stable coronary artery disease; OAC, oral anticoagulant; *n*, number; AF, atrial fibrillation; CHADS_2_, congestive heart failure, hypertension, age ≥ 75 years, diabetes mellitus, prior stroke or TIA or thromboembolism (doubled), CHA_2_DS_2_‐VASc, congestive heart failure or left ventricular dysfunction Hypertension, age ≥ 75 (doubled), diabetes, stroke (doubled)‐vascular disease, age 65–74, sex category, HAS‐BLED: hypertension, abnormal renal/liver function, stroke, bleeding history or predisposition, Labile INR, elderly, drugs/alcohol concomitantly, DOAC: direct oral anticoagulant, *n*: number.

^a^ The values are given as mean.

### 3.1 Outcomes

#### 3.1.1 Death

**Figure 2:**
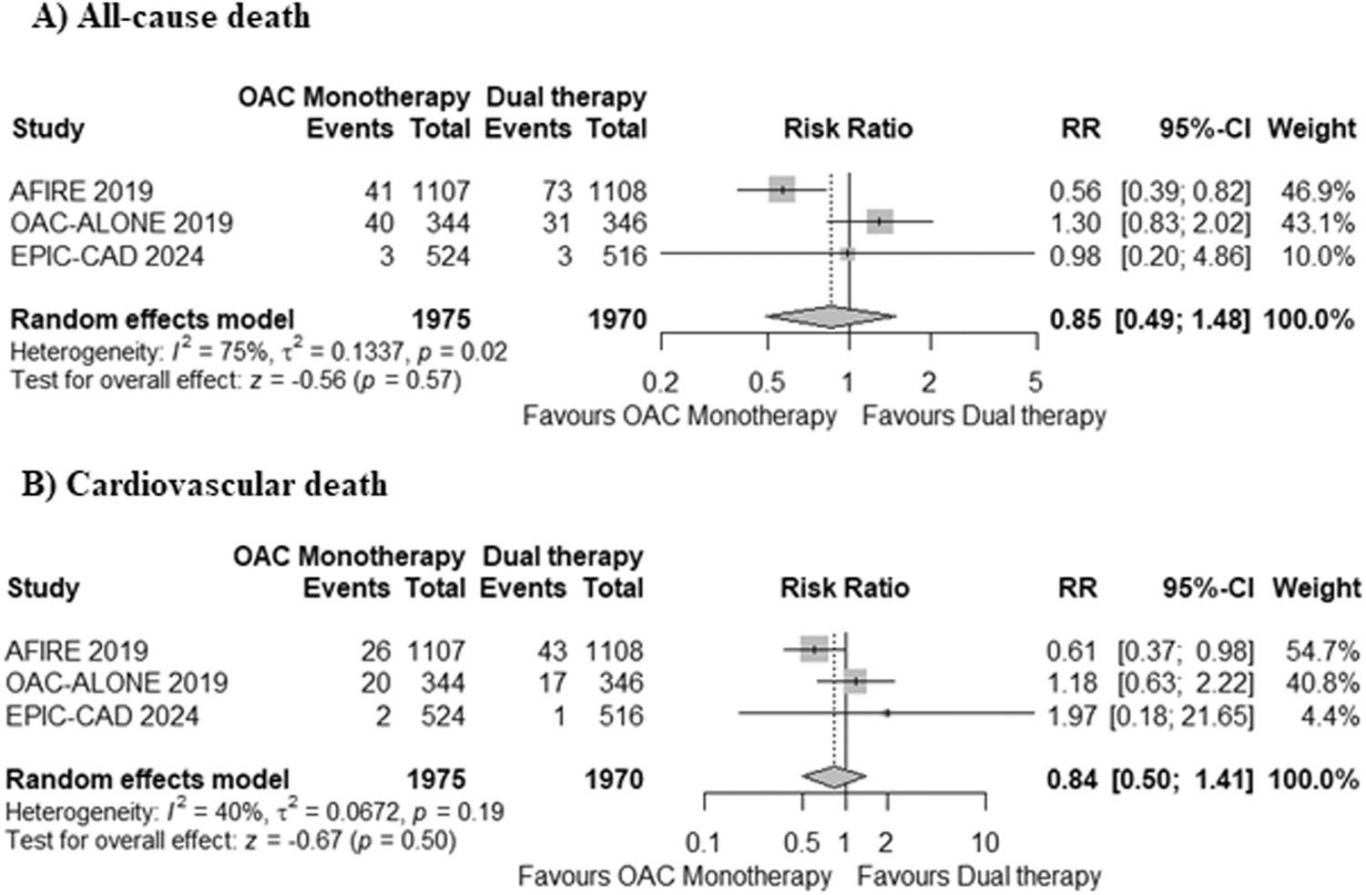
Forest plots for (A) all‐cause death, and (B) cardiovascular death. OAC, oral anticoagulant.

No statistically significant difference was observed between OAC monotherapy and dual therapy for the risk of all‐cause death (4.2% with monotherapy vs. 5.4% with dual therapy; RR: 0.85, 95% CI: 0.49–1.48, *p* = 0.57, Figure 2A) and cardiovascular death (2.4% with monotherapy vs. 3% with dual therapy; RR: 0.84, 95% CI: 0.50–1.41, *p* = 0.50, Figure 2B). A high level of between‐study heterogeneity (*I* ^2^ = 75%) was observed for all‐cause death and moderate heterogeneity (*I* ^2^ = 40%) for cardiac death.

#### 3.1.2 Major Bleeding

**Figure 3:**
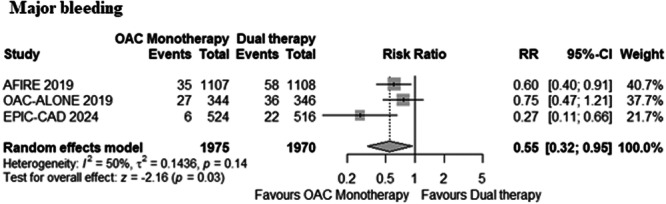
Forest plot for major bleeding. OAC, oral anticoagulant.

OAC monotherapy was associated with a significantly reduced risk of major bleeding (3.4% with monotherapy vs. 5.8% with dual therapy; RR: 0.55, 95% CI: 0.32–0.95, *p* = 0.03, Figure 3) compared to DAT. A moderate level of heterogeneity was observed (*I* ^2^ = 50%). All the included trials used the International Society on Thrombosis and Hemostasis (ISTH) criteria for reporting major bleeding.

#### 3.1.3 Stroke

**Figure 4:**
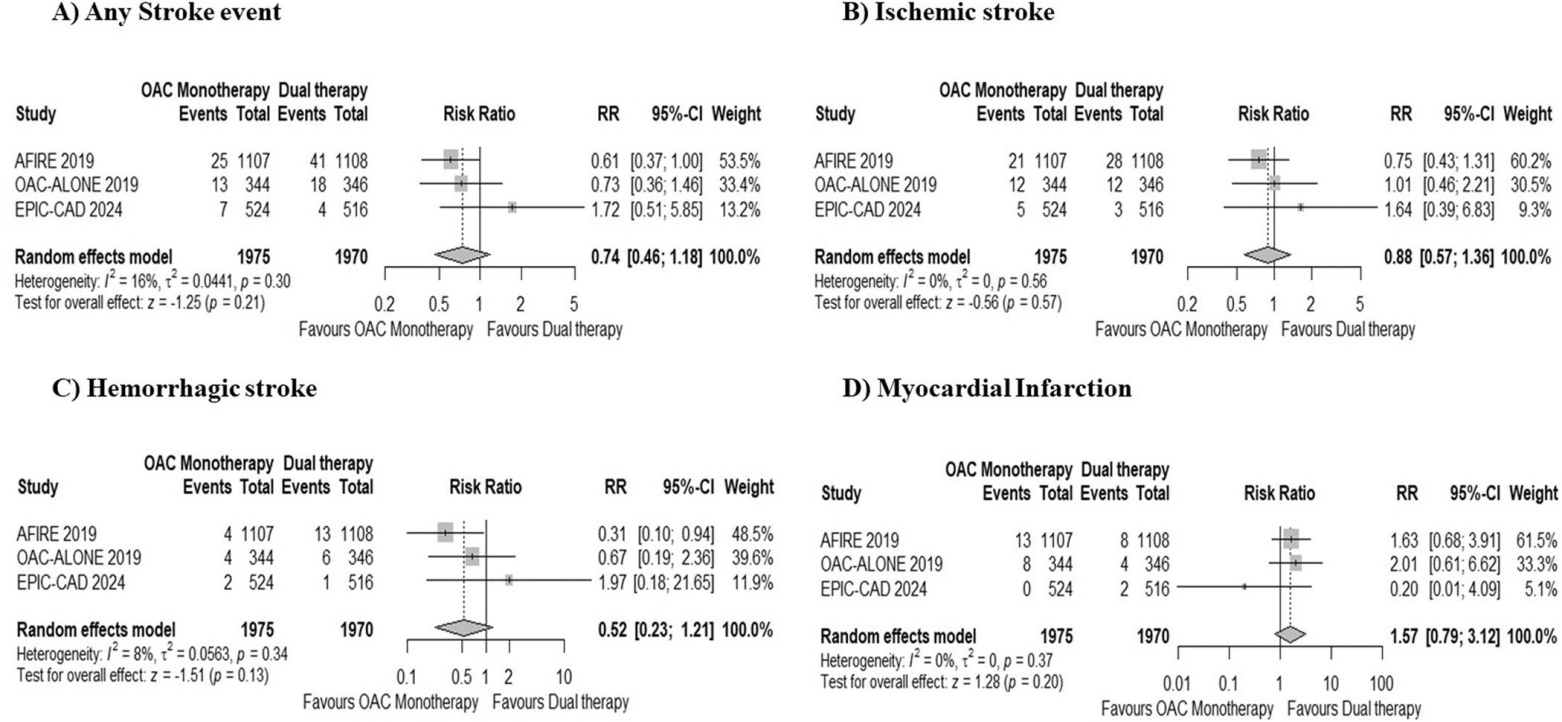
Forest plots for (A) any stroke event, (B) ischemic stroke, (C) hemorrhagic stroke, and (D) myocardial Infarction. OAC, oral anticoagulant.

The risk of any stroke event (2.2% with monotherapy vs. 3.1% with dual therapy; RR: 0.74, 95% CI: 0.46–1.18, *p* = 0.21, Figure 4A), ischemic stroke (1.9% with monotherapy vs. 2.1% with dual therapy; RR: 0.88, 95% CI: 0.57–1.36, *p* = 0.57, Figure 4B), and hemorrhagic stroke (0.5% with monotherapy vs. 1.01% with dual therapy; RR: 0.52, 95% CI: 0.23–1.21, *p* = 0.13, Figure 4C) remained comparable across both groups. Low heterogeneity was observed for all endpoints (*I* ^2^ < 25%).

#### 3.1.4 Myocardial Infarction

The pooled analysis demonstrated a nonsignificant difference between OAC monotherapy and dual therapy for the risk of myocardial infarction (1.01% with monotherapy vs. 0.01% with dual therapy; RR: 1.57, 95% CI: 0.79–3.12, *p* = 0.20, Figure 4D). No heterogeneity was observed (*I* ^2^ = 0%).

#### 3.1.5 Pooled Hazard Ratios and Sensitivity Analysis

The secondary analysis based on pooling of HRs reported by trials demonstrated similar results to the calculated risk ratios for all‐cause death, cardiac death, ischemic stroke, hemorrhagic stroke, and major bleeding (Supporting Information S1: Figures S2–S6). For myocardial infarction and any stroke event, HRs were inconsistently reported by trials and hence could not be pooled. Heterogeneity reduced to 0% by excluding AFIRE and OAC‐ALONE in the leave‐one‐out sensitivity analysis for all‐cause and cardiovascular death. By excluding EPIC‐CAD, heterogeneity reduced to 0% for major bleeding. No outlier study was identified for other outcomes (Supporting Information S1: Figures S7–S13).

## 4 Discussion

The findings of this meta‐analysis provide important insights into managing patients with AF and stable CAD. Our pooled analysis demonstrates a significantly reduced risk of major bleeding with OAC monotherapy compared to DAT. Moreover, OAC monotherapy offers comparable protection against all‐cause death, CV death, and ischemic events (myocardial infarction and stroke) in comparison to DAT. These results underscore the efficacy of oral anticoagulation alone in managing thromboembolic risk in this high‐risk population.

Our findings align with the growing body of evidence [18, 19] suggesting that the addition of antiplatelet therapy to anticoagulation, while potentially beneficial in specific circumstances, may not provide additional protective effects against ischemic events in stable CAD patients with AF. Instead, it may unnecessarily increase the risk of serious bleeding complications, which are a major concern in long‐term antithrombotic therapy [6].

The comparable rates of ischemic events and mortality between the two groups highlight that the omission of antiplatelet therapy in patients treated with anticoagulation alone does not compromise their safety in terms of thromboembolic protection. This suggests that for many patients with AF and stable CAD, OAC monotherapy may offer a more balanced approach, effectively managing both thrombotic and bleeding risks.

The clinical implications of these findings are significant. Recent literature increasingly supports the idea of simplifying antithrombotic regimens to reduce bleeding risk, particularly beyond the first year following a coronary event or intervention. The evidence from this meta‐analysis reinforces the notion that OAC monotherapy could be a safer alternative to DAT, especially in long‐term management, without sacrificing efficacy.

A notable aspect of our analysis is the inclusion of three distinct trials, each evaluating a different OAC as monotherapy—rivaroxaban, edoxaban, and either warfarin or a Direct Oral Anticoagulant (DOAC). This diversity in anticoagulant choice allows for a broader examination of the efficacy and safety profiles of different anticoagulants when used as monotherapy in this complex patient group. Despite the use of different anticoagulants, the consistent outcomes in reducing major bleeding across these trials suggest that the benefits of OAC monotherapy may be a class effect rather than being restricted to any specific agent. This finding is particularly significant because it supports the flexibility in anticoagulant choice, allowing clinicians to tailor therapy based on patient‐specific factors such as renal function, drug interactions, and patient preference. However, it is important to consider the differences in the pharmacological profiles of these anticoagulants. For instance, both rivaroxaban and edoxaban offer the advantage of fixed dosing without the need for routine monitoring [20], unlike warfarin, which requires regular international normalized ratio (INR) monitoring due to its variable response and numerous drug interactions [21]. Future research should continue to explore the comparative effectiveness of different anticoagulants in this setting.

This meta‐analysis adds to the literature by integrating results from a newly published clinical trial [10], enhancing the robustness of the evidence base. However, it has certain limitations. This meta‐analysis is based on study‐level data, and the duration of DAT was not available. A dedicated meta‐analysis based on individual patient data may provide further information to confirm the optimal antithrombotic regimen in this complex field. It is important to acknowledge that while these findings are compelling, they should be interpreted within the context of the individual patient's clinical profile, considering factors such as their bleeding risk, the nature of their CAD, and the time elapsed since any coronary interventions. Moreover, it should be considered that the point estimate for MI was > 1.0 with wide confidence intervals. Therefore, additional trial data would be informative.

## 5 Conclusion

In conclusion, this analysis supports the use of OAC monotherapy as a viable and potentially preferable option for patients with AF and stable CAD compared to DAT, offering similar protection against thromboembolic events with a significantly reduced risk of major bleeding. These findings may guide clinicians to make more informed decisions regarding antithrombotic therapy in this complex patient population, emphasizing the importance of a tailored approach to treatment.

## Author Contributions

**Mushood Ahmed:** conceptualization, data curation, and project administration, formal analysis, methodology, and software, writing the original draft, writing, reviewing, and editing, visualization, and validation. **Areeba Ahsan:** formal analysis of data, formal analysis, methodology, and software, writing the original draft. **Aimen Shafiq:** formal analysis, methodology, and software, writing the original draft. **Raheel Ahmed:** writing, reviewing, and editing. **Mahboob Alam:** formal analysis of data. **Pierre Sabouret:** supervision, writing, reviewing, and editing. **Jamal S. Rana:** conceptualization, data curation, and project administration, writing, reviewing, and editing. **Gregg C. Fonarow:** supervision, writing, reviewing, and editing.

## Ethics Statement

The authors have nothing to report.

## Conflicts of Interest

Dr. Gregg C. Fonarow reported receiving personal fees from Abbott, Amgen, AstraZeneca, Bayer, Boehringer Ingelheim, Cytokinetics, Eli Lilly, Johnson & Johnson, Medtronic, Merck, Novartis, and Pfizer outside the submitted work.

## Supporting information

Supporting information.

## Data Availability

The data that support the findings of this study are available from the corresponding author upon reasonable request.
