# Supplementary material for: Meta‐Analysis Comparing Oral Anticoagulant Monotherapy Versus Dual Antithrombotic Therapy in Patients With Atrial Fibrillation and Stable Coronary Artery Disease
Source: Clin Cardiol. 2024 Oct 7;47(10):e70026. doi: 10.1002/clc.70026 (PMC11457041; doi:10.1002/clc.70026)

**Supplementary Appendix**

Table S1: Detailed search strings for databases used

| Database | Search string | Records |
| --- | --- | --- |
| PubMed/MEDLINE | (("AF"[All Fields] OR "atrial fibrillation"[All Fields]) AND ("coronary stenting"[All Fields] OR "coronary angioplasty"[All Fields] OR "PCI"[All Fields] OR "percutaneous coronary intervention"[All Fields] OR ("stent s"[All Fields] OR "stentings"[All Fields] OR "stents"[MeSH Terms] OR "stents"[All Fields] OR "stent"[All Fields] OR "stented"[All Fields] OR "stenting"[All Fields]) OR ("stent s"[All Fields] OR "stentings"[All Fields] OR "stents"[MeSH Terms] OR "stents"[All Fields] OR "stent"[All Fields] OR "stented"[All Fields] OR "stenting"[All Fields]) OR "drug-eluting stent"[All Fields] OR "DES"[All Fields] OR ("bull methodol sociol"[Journal] OR "biomed mass spectrom"[Journal] OR "bms"[All Fields]) OR "bare metal stent"[All Fields] OR "acute coronary syndrome"[All Fields]) AND ("antithrombotic therapy"[All Fields] OR ("2 deoxythymidylyl 3 5 2 deoxyadenosine"[Supplementary Concept] OR "2 deoxythymidylyl 3 5 2 deoxyadenosine"[All Fields] OR "dapt"[All Fields]) OR "dual antiplatelet therapy"[All Fields] OR ("clopidogrel"[MeSH Terms] OR "clopidogrel"[All Fields] OR "clopidogrel s"[All Fields]) OR ("ticagrelor"[MeSH Terms] OR "ticagrelor"[All Fields] OR "ticagrelor s"[All Fields]) OR ("prasugrel hydrochloride"[MeSH Terms] OR ("prasugrel"[All Fields] AND "hydrochloride"[All Fields]) OR "prasugrel hydrochloride"[All Fields] OR "prasugrel"[All Fields] OR "prasugrel s"[All Fields]) OR "P2Y12 inhibitor"[All Fields] OR "triple therapy"[All Fields] OR "antithrombotic drugs"[All Fields] OR ("antiplatelet"[All Fields] OR "antiplatelets"[All Fields]) OR "oral anticoagulant"[All Fields] OR "VKA"[All Fields] OR ("n 4 oleylcytosine arabinoside"[Supplementary Concept] OR "n 4 oleylcytosine arabinoside"[All Fields] OR "noac"[All Fields]) OR "DOAC"[All Fields] OR ("dabigatran"[MeSH Terms] OR "dabigatran"[All Fields] OR "dabigatran s"[All Fields]) OR ("apixaban"[Supplementary Concept] OR "apixaban"[All Fields] OR "apixaban s"[All Fields]) OR ("edoxaban"[Supplementary Concept] OR "edoxaban"[All Fields]) OR ("rivaroxaban"[MeSH Terms] OR "rivaroxaban"[All Fields] OR "rivaroxaban s"[All Fields]))) AND (randomizedcontrolledtrial[Filter]) | 116 |
| Embase | (af OR 'atrial fibrillation'/exp OR 'atrial fibrillation') AND ('coronary stenting'/exp OR 'coronary stenting' OR 'coronary angioplasty'/exp OR 'coronary angioplasty' OR 'percutaneous coronary intervention'/exp OR 'percutaneous coronary intervention' OR pci OR stenting OR 'stent'/exp OR 'stent' OR 'drug-eluting stent'/exp OR 'drug-eluting stent' OR des OR bms OR 'bare metal stent' OR 'acute coronary syndrome'/exp OR 'acute coronary syndrome') AND ('antithrombotic therapy'/exp OR 'antithrombotic therapy' OR dapt OR 'dual antiplatelet therapy'/exp OR 'dual antiplatelet therapy' OR 'clopidogrel'/exp OR clopidogrel OR ticagrelor OR prasugrel OR 'p2y12 inhibitor'/exp OR 'p2y12 inhibitor' OR 'triple therapy'/exp OR 'triple therapy' OR 'antithrombotic drugs' OR 'antiplatelet agent'/exp OR antiplatelets OR 'oral anticoagulant'/exp OR 'oral anticoagulant' OR vka OR noac OR doac OR dabigatran OR apixaban OR edoxaban OR rivaroxaban) AND 'randomized controlled trial topic'/de | 852 |
| Cochrane Library | (AF OR "atrial fibrillation") AND ("coronary stenting" OR "coronary angioplasty" OR PCI OR "percutaneous coronary intervention" OR stenting OR stent OR "drug-eluting stent" OR DES OR BMS OR "bare metal stent" OR "acute coronary syndrome") AND ("antithrombotic therapy" OR DAPT OR "dual antiplatelet therapy" OR clopidogrel OR Ticagrelor OR Prasugrel OR "P2Y12 inhibitor" OR "triple therapy" OR "antithrombotic drugs" OR antiplatelets OR "oral anticoagulant" OR VKA OR NOAC OR DOAC OR dabigatran OR apixaban OR edoxaban OR rivaroxaban) | 418 |

Figure S1: PRISMA flowchart showing the screening and study selection process


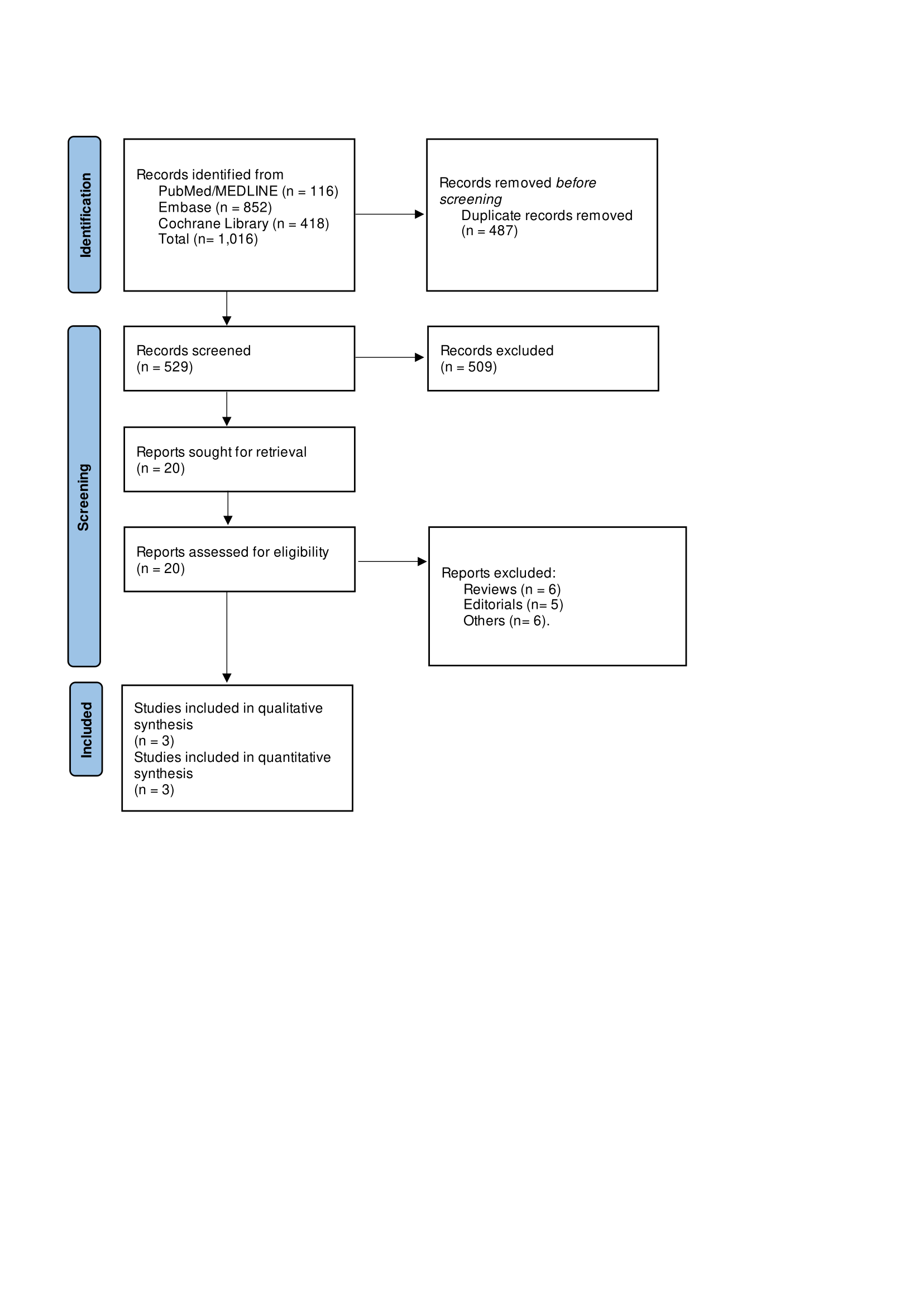


Table S2: Details of baseline characteristics of included patients

| **Trial** | **Year** | **Diabetes-n (%)** | | **Dyslipidemia-n (%)** | | **Current smoker-n (%)** | | **Previous myocardial infarction-n (%)** | | **Prev Stroke-n (%)** | | **Heart failure n(%)** | | **BMI (Mean ± SD)** | | **Type of stent- DES- n (%)** | |
| --- | --- | --- | --- | --- | --- | --- | --- | --- | --- | --- | --- | --- | --- | --- | --- | --- | --- |
|  |  | **OAC Monotherapy** | **Dual therapy** | **OAC Monotherapy** | **Dual therapy** | **OAC Monotherapy** | **Dual therapy** | **OAC Monotherapy** | **Dual therapy** | **OAC Monotherapy** | **Dual therapy** | **OAC Monotherapy** | **Dual therapy** | **OAC Monotherapy** | **Dual therapy** | **OAC Monotherapy** | **Dual therapy** |
| **AFIRE** | **2019** | 461 (41.6) | 466 (42.1) | 781 (70.6) | 757 (68.3) | 146 (13.2) | 146 (13.2) | 384 (34.7) | 393 (35.5) | 148 (13.4) | 175 (15.8) | 389 (35.1) | 399 (36.0) | 24.5±3.7 | 24.5±3.7 | 500/723 (69.2) | 477/721 (66.2) |
| **OAC-ALONE** | **2019** | 152(44.2) | 138(39.9) | 294(85.5) | 298(86.1) | 27(7.9) | 23(6.7) | 129(37.5) | 500/723 (69.2) | 500/723 (69.2) | 49(14.2) | 140(40.7) | 151(43.6) | 24.3±3.4 | 24.4±3.4 | 246(71.7) | 240(70.6) |
| **EPIC-CAD** | **2024** | 224 (42.7) | 197 (38.2) | 490 (93.5) | 482 (93.4) | 37 (7.1) | 50 (9.7) | 79 (15.1) | 246(71.7) | 246(71.7) | 77 (14.9) | 96 (18.3) | 109 (21.1) | 25.3±3.3 | 25.4±3.3 | 251/308 (81.5) | 267/318 (84.0) |

AFIRE: Atrial Fibrillation and Ischemic Events with Rivaroxaban in Patients with Stable Coronary Artery Disease, OAC-ALONE: The Optimizing Antithrombotic Care in Patient with Atrial Fibrillation and Coronary Stent, EPIC-CAD: Edoxaban versus Edoxaban with Antiplatelet Agent in Patients with Atrial Fibrillation and Chronic Stable Coronary Artery Disease, OAC: oral anticoagulant, BMI: body mass index, n: number

Figure S2: Forest plot showing pooled HR for all-cause death


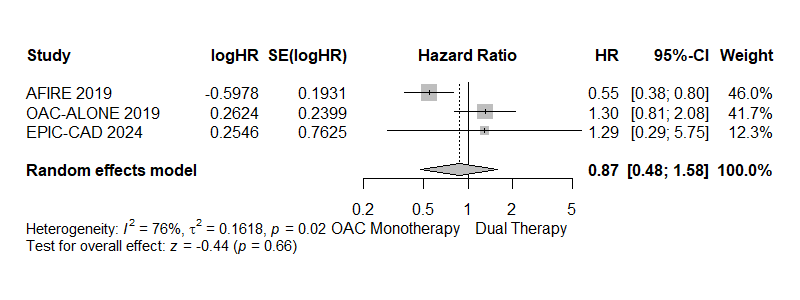


Figure S3: Forest plot showing pooled HR for cardiovascular death


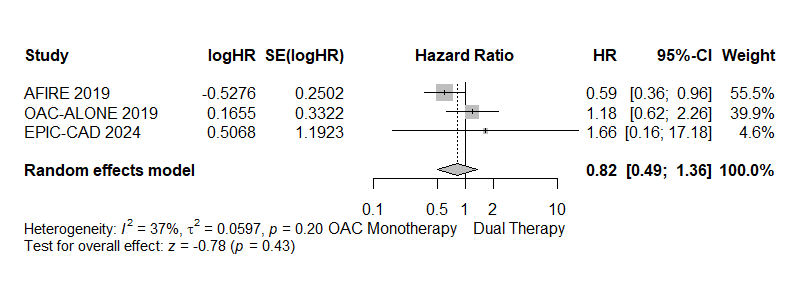


Figure S4: Forest plot showing pooled HR for ischemic stroke


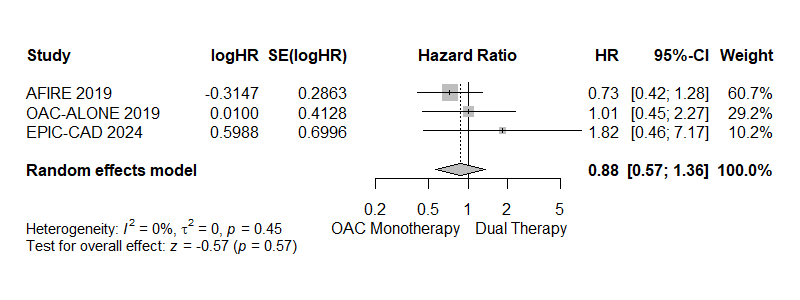


Figure S5: Forest plot showing pooled HR for hemorrhagic stroke


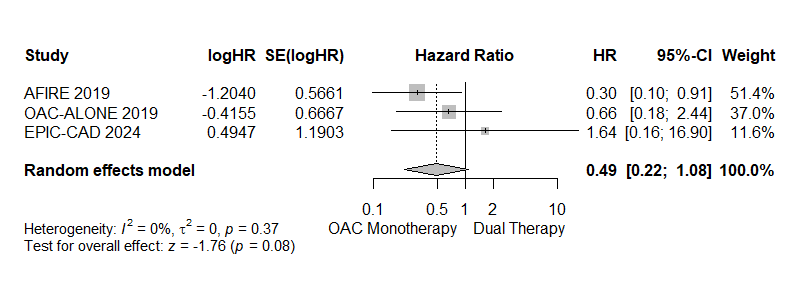


Figure S6: Forest plot showing pooled HR for major bleeding


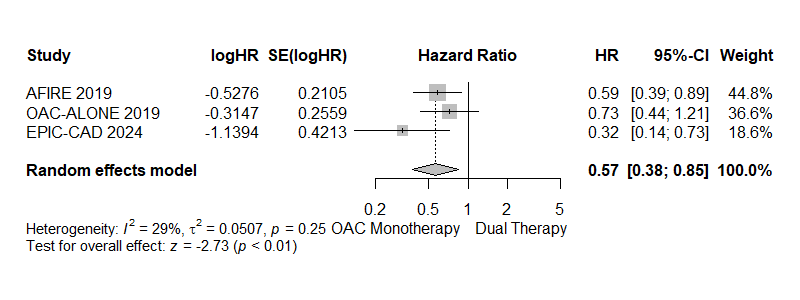


Figure S7: Leave-on-out sensitivity analysis for all-cause death


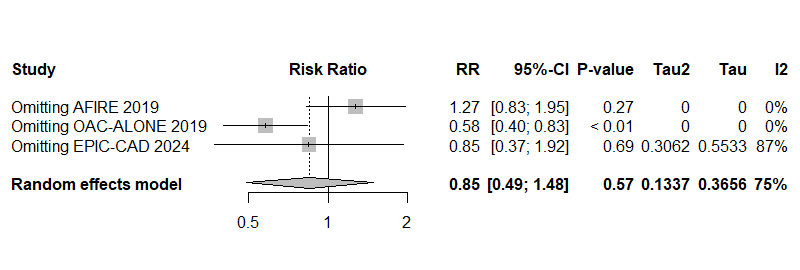


Figure S8: Leave-on-out sensitivity analysis for cardiovascular death


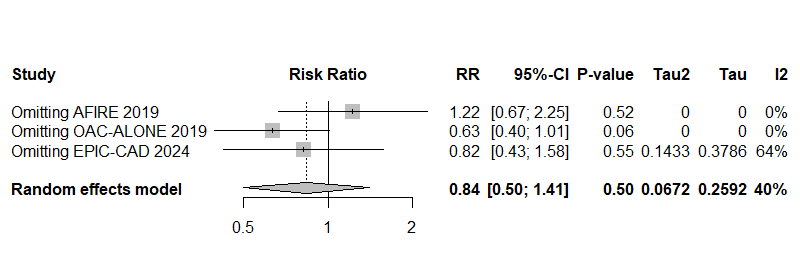


Figure S9: Leave-on-out sensitivity analysis for major bleeding


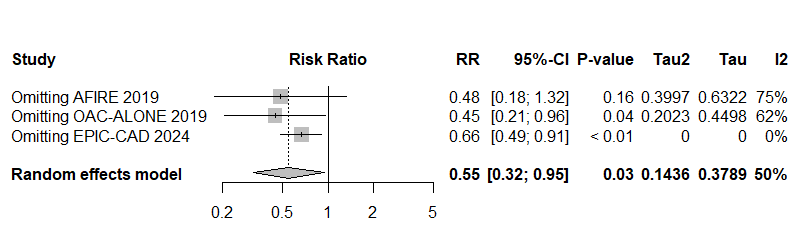


Figure S10: Leave-on-out sensitivity analysis for any stroke event


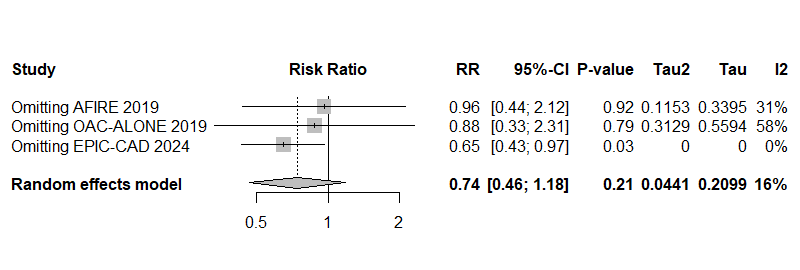


Figure S11: Leave-on-out sensitivity analysis for ischemic stroke


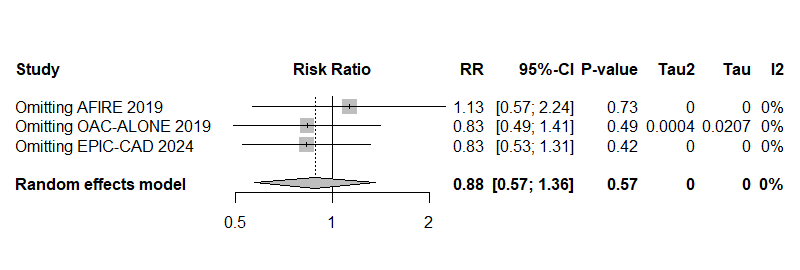


Figure S12: Leave-on-out sensitivity analysis for hemorrhagic stroke


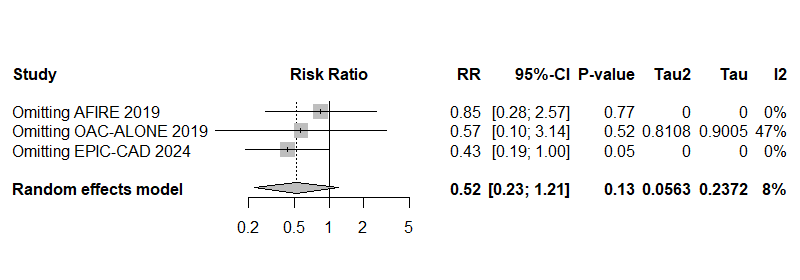


Figure S13: Leave-on-out sensitivity analysis for myocardial infarction


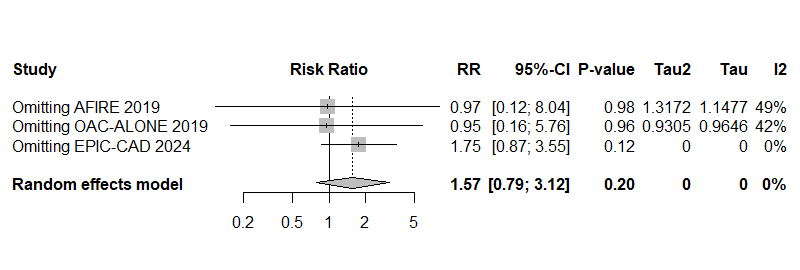

Supplement: Supplementary file 1 — Supporting information. [file CLC-47-e70026-s001.docx]
